# Engaging pregnant women in observational research: a qualitative exploratory study

**DOI:** 10.1186/s12884-018-1966-z

**Published:** 2018-08-16

**Authors:** Evelyne Muggli, Helen Curd, Cate Nagle, Della Forster, Jane Halliday

**Affiliations:** 1 Murdoch Childrens Research Institute, 50 Flemington Rd, Parkville, VIC 3052 Australia; 2 Department of Paediatrics, The University of Melbourne, 50 Flemington Rd, Parkville, VIC 3052 Australia; 3 Monash Health Genetics Department, Monash Medical Centre, 246 Clayton Rd, Clayton, VIC 3168 Australia; 4 Centre for Nursing and Midwifery Research, James Cook University, 1 James Cook Dr, Townsville, QLD 4811 Australia; 5 Townsville Hospital and Health Service, 100 Angus Smith Dr, Douglas, QLD 4814 Australia; 6 Judith Lumley Centre, School of Nursing and Midwifery, La Trobe University, Plenty Rd & Kingsbury Dr, Bundoora, VIC 3086 Australia; 7 The Royal Women’s Hospital, Grattan St, Parkville, VIC 3052 Australia

**Keywords:** Recruitment, Perinatal research, Alcohol use in pregnancy

## Abstract

**Background:**

Recruitment of pregnant women to population health research can be challenging, especially if the research topic is sensitive. While many pregnant women may be inherently interested in research about pregnancy, there is the possibility that the nature and timing of the project may give rise to anxiety in some women, especially if the topic is sensitive or it brings about new awareness of potential pregnancy complications. Research staff undertaking recruitment need to be skilled at strategies to manage the environment, and have well developed communication and interpersonal skills to explain and promote the study and facilitate each woman’s informed decision-making regarding participation.

However, the skills needed by recruitment staff to successfully engage pregnant women with a research topic are not well understood. This study aimed to address this evidence gap by providing insight into the dynamics between a pregnant woman and recruitment staff at the time of the offer to participate in an observational study about alcohol use in pregnancy.

**Methods:**

Naturalistic inquiry guided a qualitative exploratory descriptive approach. Experienced recruitment staff from the Asking Questions about Alcohol in Pregnancy (AQUA) study (Muggli et al., BMC Pregnancy Childbirth 14:302, 2014) participated in individual semi-structured interviews and were asked about their experiences and approaches to engaging pregnant women. Interviews were transcribed verbatim and analysed using inductive content analysis.

**Results:**

Pregnant women brought with them an inherent interest or disinterest in alcohol research, or in research in general, which formed the basis for engagement. Women responded favourably to the invitation to participate being delivered without pressure, and as part of a two-way conversation. Engagement with a sensitive topic such as alcohol use in pregnancy was facilitated by a non-judgmental and non-targeted approach. Influences such as privacy, distractions, partner’s opinion, time factors and level of clinical support either facilitated or hindered a woman’s engagement with the research.

**Conclusions:**

These results provide an in-depth explanation of barriers and enablers to recruitment of pregnant women in antenatal clinics to studies that may inform strategies and the training of recruitment staff.

## Background

Recruitment of pregnant women to population health research can be challenging, especially if the research topic is sensitive. Factors that influence recruitment have been described, such as the resource requirements to maximise recruitment [1]; sampling logistics, including time constraints [2–6]; and participant demographics [1, 3]. Reasons for women’s non-participation include research procedures, such as biospecimen collection [4, 7–12]. It is important that the research staff undertaking recruitment are skilled at strategies to manage the environment, and that they have well developed communication and interpersonal skills to explain and promote the study and facilitate each woman’s informed decision making regarding participation.

Pregnant women who participate in research are usually motivated by their interest in science and learning more about their pregnancy [13, 14]. They often represent women from higher educational and socioeconomic backgrounds, or women who are less ethnically diverse than the overall population [1, 3], which can limit the generalisability of some studies. Research staff need to be able to explain studies to women with a variety of health literacy levels.

While many pregnant women may be inherently interested in research about pregnancy, there is the possibility that the nature and timing of the project itself may give rise to anxiety in some women, especially if the topic is sensitive or it brings about new awareness of potential pregnancy complications. Gaining trust and genuine interest from women is critical to a study’s success, and requires multiple approaches.

Participation rates are increased with multi-faceted methods such as networking with clinicians at recruitment sites, supplying study brochures and posters, having an online presence and direct participant contact [1]. However, not much is known about the skills needed by recruitment staff to successfully engage pregnant women with the research topic, and how this may influence participation. The aim of this qualitative study was to gain insight into women’s engagement with observational research from the perspectives of recruiting staff who collectively approached over 3000 women for the study. This paper describes the views and experiences of staff recruiting pregnant women into the Asking Questions about Alcohol in Pregnancy (AQUA) study [15].

## Methods

### Study design

To explore our aim, a naturalistic inquiry using a qualitative exploratory descriptive research approach was employed [16, 17]. Naturalistic inquiry underpins qualitative research to the degree that it involves people in everyday situations and the research develops naturally. Data was collected using semi-structured interviews of recruitment staff of the AQUA study.

Recruiting staff were asked to describe their experiences or understanding of a given topic to elucidate the meaning they attached to the topic. In this study, because no relevant theoretical framework was identified, naturalistic inquiry provided an ideal approach to gain insight into pregnant women’s engagement in observational research from the perspective of midwives and nurses.

Participation in this study was offered via email by the AQUA study project manager (EM) to nine of 13 past and present AQUA study recruitment staff for whom current contact details were available. The interviews were conducted in Melbourne, Victoria, Australia between July and September 2012, at a time and venue convenient to the recruiting staff member. Written consent was obtained including consent for the interviews to be audiotaped. Interviews were undertaken by a Master of Genetic Counselling student, independent of the AQUA study and with no line management to the recruiting staff. A schedule covering four key areas; first approach; broaching the topic of alcohol in pregnancy; specific influences around engagement; and impact of the woman’s knowledge of the consequences of alcohol use in pregnancy was used. Interviews were transcribed verbatim by HC and recruiting staff names were replaced with pseudonyms; non-verbal communication, such as the degree of spontaneity and recruiting staff involvement, was added to the transcript from field notes where appropriate.

### Data analysis

Basic demographic data were collected for each recruiting staff member, including qualifications and research experience. In keeping with a naturalistic inquiry approach, transcripts were analysed using inductive content analysis [18]. Content analysis is particularly useful to systematically and objectively identify specific messages in any type of social communication. Content analysis establishes the existence and frequency of concepts through inclusion or exclusion of content according to consistently applied criteria relevant to the research aims [18].

**Fig. 1:**
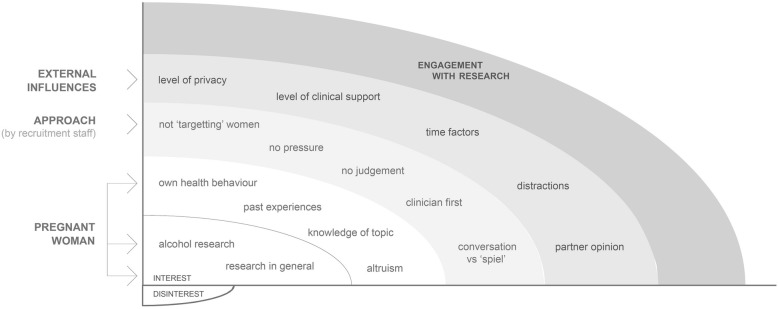
Influences on pregnant women’s engagement with observational research

Analysis involved HC and EM repeatedly and independently reading the transcripts, while coding and annotating the text in the margins with headings, which represented manageable content categories. Comparisons for coding reliability was a process of discussion and deliberation of categories and connections between them. A process of selective reduction then produced an agreed analysis matrix, which consisted of hierarchical flow charts to represent each heading and connections [18]. Data were then abstracted into this matrix in a dynamic process by further reviewing and refining headings with similar responses. These formulated categories became the final framework used to report results (see Fig. 1). Each category was named using a term that was ‘content-characteristic’ [18].

## Results

Seven recruitment staff responded to our invitation, six of whom were still recruiting for the AQUA study at the time. Five recruiting staff were registered midwives (anonymised as midwife 1–5 in quotes), one a registered nurse and another a Science Graduate with a Master’s in Public Health (anonymised as Other professional 1 and 2 in quotes). All were employed as recruitment staff with the AQUA study (for a range of 4 to 12 months), they collectively covered five different recruitment sites, and individuals recruited between 52 and 821 women. Three recruiting staff had previous research experience in a clinical setting. Interviews took between 30 and 90 min to complete.

Content analysis by the investigators resulted in three categories influencing a pregnant woman’s level of engagement with alcohol research. Firstly, pregnant women brought with them an inherent interest or disinterest in the research topic, which formed the basis for engagement. Secondly, a specific set of competencies and approaches emerged when the recruitment staff described how they navigated their conversations with the women. Thirdly, there were external impacts, beyond the control of the recruitment staff, which either facilitated or hindered a woman’s engagement with the research. Up to six keywords further explained each category to increase understanding and generate knowledge of the topic. Figure 1 shows the conceptual framework, which is discussed below.

### The disposition of the pregnant woman towards research

#### Inherent disinterest

Pregnant women approached in the clinic waiting rooms very quickly tended to display an inherent interest, or disinterest in this study, or in research in general. Recruitment staff related how they learned to recognise early that some women were not interested, and did not offer any opportunity for engagement or recruitment to the study. Reasons for this were not always given, but often related to distractions such as other children being present and general comments such as “too busy” or “not interested” [15]. In keeping with the ethical principles of respect and justice [19] recruiting staff were required to invite every eligible woman to the study. They identified the signs of a likely non-acceptance either through the woman’s body language or through a closed-off response as soon as they approached her.

*“As the recruiting went on, I always had a sense, before I even walked up to a woman, whether they were open to helping with the research.” (Midwife 1)*

*“I found fairly much that if they weren’t interested, you could almost get a feel for it quickly about the ones who were probably going to say no.” (Midwife 2)*

*“If somebody said they weren’t interested, I’d sort of thank them for their time and I’d never push the issue, I just left them; if they said no, then they said no.” (Midwife 3)*

#### Inherent interest

Women who engaged in a dialogue about the research fell into two categories. Firstly, many women were interested in the specific topic of research about the effects of alcohol on the unborn child. Recruiters explained that women’s decisions to participate appeared to be influenced by their *own alcohol use behaviour,* their *experiences with alcohol use behaviour of others*, such as family and friends and their *knowledge* of the topic; women’s knowledge of the topic often related to the uncertainties around harm from occasional social drinking. Together, these topics influenced women’s personal decisions around alcohol use in pregnancy as well as their interest in study participation and allowed staff to involve them with different aspects of the research.

“*They were very open, especially because they’re often told not to drink, or they know pregnancy and alcohol don’t really mix, but they may have had friends who do [drink]” (Other professional 1).*

*“You get a lot of comments saying ‘oh you know, my mother drank or you know, whoever, and I turned out okay’ and things like that” (Midwife 3)*

*“They’d say ‘yeah, I just discussed that with my girlfriend or in play group and we had discussed alcohol; some of them would go on and tell stories that some of their friends had drunk alcohol through their previous pregnancy and were doing the same thing again’” (Midwife 1).*

Secondly, many pregnant women showed interest in the research for *altruistic reasons,* which was not specific to the topic. Nonetheless, this interest opened up an avenue of conversation about the project, allowing recruiters to explain how participation may contribute to an increased knowledge for other pregnant women.

*“I found that they thought that hopefully in the future, they would be helping somebody else with a dilemma that they had faced themselves [a social drink in pregnancy].” (Midwife 2)*

*“Most of them would say; “I think it’s quite an important area to do more research into” and “it affects pretty much everybody when they’re pregnant”.” (Midwife 3)*

*“I said that by them being part of the study, with 2000 women, so it wasn’t a small group, it was a large group of women, that they would receive information after the project had finished, and that would empower them with knowledge to pass on to others that were planning pregnancy. I think that helped them feel like they were doing something really worthwhile to help children, and have knowledge to pass on to other mothers. Women with babies love to pass on lots of knowledge.” [Midwife 1]*

#### Approaches

In addition to the importance of emphasising general information, such as confidentiality, privacy and the voluntary nature of participation, recruitment staff described many other aspects of their approaches to women in the clinic, which reflected a skill set and understanding of the research beyond administrative requirements. These approaches were adapted, not only for individual women, but also over time as the recruitment staff’s skills deepened.

The best way to begin the conversation with women was mostly described as ensuring women *did not feel targeted* for the research and the invitation to participate was delivered *without pressure*. Even though the women were not asked to disclose whether they had been drinking in pregnancy, recruiters thought it important to be completely *non-judgemental* in their approach to the topic of drinking in pregnancy, and to relate to the women at a personal level.

*“And making sure that when I approached the ladies that they knew I just approached everybody less than 19 weeks, that was a very big thing. I think I sort of worked out that it was important, so they didn’t think they were being targeted for any reason.” [Midwife 2]*

*“Because there’s lots of women sitting in the clinic, you might have 50 women sitting there of all different sizes and gestations. You didn’t want women to have a sense that they were being singled out.” [Midwife 3]*

*“And I do tell them that they’re free to withdraw at any time and change [their] mind about anything.” [Other professional 1]*

With increasing experience, recruitment staff learned to move away from their rehearsed speeches and embed the invitation in a more personalised, relaxed *conversation*, which helped women to be more open about the research. The midwife recruiters felt it was helpful to explain first that *they were midwives* before introducing the research, as this seemed to make women more comfortable to engage in the conversation. For others, they felt that being present with the apparent approval of clinic staff legitimised their role and that of the research in the maternity clinic.

*“I introduced myself and said I was part of the study, I used the fact that I was a midwife and worked at the hospital, so I think that made them feel comfortable rather than someone that was randomly off-site.” [Midwife 1]*

*“I would go sit next to them, tell them who I am, where I am from, and what I’m doing; ‘I’m a midwife working in research.’*” *[Midwife 4]*

*“It wasn’t like pressing play and it’s recorded a message, you know, I did change my feel at times, but I pretty much said exactly the same thing but in different ways.” [Midwife 3]*

*“I didn’t have problems making people feel comfortable when they were giving me the answers, but maybe because I didn’t feel uncomfortable asking them. And I think that’s the key.” [Midwife 4]*

*“They’d seen me come out from behind the counter, so I clearly had some legitimacy...I had the folder in my arm, I looked professional and yet approachable, so all of those things were as important as what I actually said.” [Other professional 2]*

#### External impacts on engagement

Recruitment staff discussed many influences, which were out of their control, but affected women’s ability to engage with the project, either positively or negatively. These external impacts can be depicted as environmental or specific to the woman. Firstly, a leading topic related to the waiting room environment. Larger clinics tended to be extremely busy, making it difficult for quiet private conversations. Often there would not be any empty seats next to eligible women and sometimes women sat on the floor while waiting for their appointments. This hindered recruitment efforts considerably because recruiters were conscious of the *lack of privacy* when talking to women about a potentially sensitive topic. Some clinics provided private rooms for recruiters, which was extremely useful in overcoming these issues.

*“The room doesn’t have enough seats a lot of the time for the ladies to sit down. Sometimes I found, even just to be able to talk to the ladies, I’d be squatting down on the floor next to them, while they were on a seat, or we’d be standing up over against a wall. So, it was really difficult.” [Midwife 2]*

*“We’d go out and call the women’s names and there would be pregnant women sitting on the floor. There was not enough seats, not even enough seats for us to actually sit down and get to the same level of the women we’re recruiting.” [Midwife 3]*

Secondly, *support by clinical and administrative staff* was crucial to success in engaging women with the research. On occasion, recruitment staff felt they were a nuisance, adding to the busy clinical workload, although more often, reception staff assisted recruiters to identify eligible women from their booking lists and clinical midwives introduced the recruiter during antenatal visits. This legitimised the presence of the recruiters, which in turn increased the engagement of women.

*“The midwives were actually really helpful. A couple of midwives here and there, they weren’t that helpful, But [mostly] they were saying for instance; ‘have you heard about this study? [Midwife 4] going to talk to you after.” [Midwife 4]*

*“Most of the time the midwives were too busy. We were sort of hoping they would help us initially, but it was clear quite early that they were really busy and that they didn’t have time to come and tell us that, or identify any [eligible women] to us.” [Midwife 3]*

Next, if women were called into their appointments with little waiting time, or while talking to recruiters, it was difficult to complete recruitment as women could be difficult to locate afterwards or recruiters would then be in conversation with another potential participant. This *time factor* proved a substantial issue, which could not be addressed as it was intrinsic to how the clinics operated.

*“Frequently they would arrive, sit down and would be called in straight-away, which meant that our window wasn’t open.” [Other professional 2]*

*“Time was always an issue, because the first appointment takes about an hour. So, completing the questionnaire meant doing it before or after, and 5 minutes of recruitment is 5 minutes that you really don’t have.” [Midwife 4]*

Secondly, recruiters talked about how *distractions* such as the presence of small children sometimes changed how women engaged with their approach. Sometimes women needed to attend to their toddlers, taking up their available attention.

*“I think some of the ones who had toddlers, they probably would’ve been interested if they weren’t so busy with another child. That was probably quite a common reason I got; ‘Look I’m really busy at the moment’”. [Midwife 2]*

*“I don’t think there have been any that have been really difficult to approach, other than the ones that are very distracted with little kids.” [Other professional 1]*

Other times, when partners were present, their opinion occasionally differed from the woman’s, which quickly ended any conversation about taking part in the research.

*“Once again, you picked up the non-verbal body language that the partner was not impressed and didn’t want his wife or partner being part of a study regarding alcohol… The way they sat, particularly the men, I would say their body language became a bit assertive. They’d either sit forward on their seat or some of them would initially engage, as in eye to eye contact but then they’d sit back in their chair and not engage the eye to eye contact.” [Midwife 1]*

*“I did get the feeling that there were a few ladies who would have participated, but didn’t, because their husbands or fathers didn’t want them to.” [Midwife 5]*

## Discussion

This interview study presents approaches and experiences of practised recruitment staff on engaging pregnant women in observational research, specifically alcohol research, with a view to increasing the understanding of the dynamics of participant recruitment. Results provide useful information for researchers planning to recruit pregnant women, which will inform the training of recruitment staff.

Our research complements that of others, which document influences on recruitment of pregnant women, its qualitative design providing additional insight on how to maximise engagement of potential participants. For example, face-to face recruitment and multi-faceted approaches, such as those used by the AQUA study, have already been demonstrated as effective strategies [1, 20]. Our study adds that identifying the nature of the woman’s interest early assisted with conversations about the research project and tailoring the information provided. These dialogues made it easier to engage women than a standard, one-for-all rehearsed invitation to participate. A qualitative interview study of 18 pregnant women who declined participation in a clinical trial confirms that the sharing of information at recruitment is important in how an invitation is received [8]. Further, there were a number of effective approaches used by all recruitment staff in our study. These related to ensuring women did not feel singled out for the research, nor judged for their opinions or actions around drinking in pregnancy.

Our findings confirm previous claims that pregnant women who are willing to engage in research are often altruistically motivated [13, 14], and add that some women bring with them a fundamental disinterest in research or they grapple with distractions, such as the presence of children, both which almost always preclude recruitment.

There were also some factors not directly related to the interactions between eligible women and researchers, but which influenced the potential of recruitment. For example, a good working relationship with clinic staff was seen to legitimise the presence of the researchers and imbuing trust in pregnant women attending clinic appointments. Supportive study sites provided opportunities for private conversations and allocated time (e.g. in dedicated clinical rooms), which greatly facilitated engagement. This highlights the importance for taking the time to build a strong organisational relationship to create buy-in from study sites prior to commencement of the research.

There are some limitations to be considered when interpreting our findings in the context of other situations. Firstly, our study infers the influences on engagement of pregnant women with observational research from the experiences of recruitment staff. These may be coloured by the individual approaches used and it is possible that there are other methods to engage pregnant research participants. However, our recruitment staff collectively spoke to thousands of women across several maternity clinics over the course of 12 months, during which they gained considerable expertise, encountering numerous pregnant women from diverse backgrounds. Secondly, our research study related to alcohol consumption in pregnancy, a potentially sensitive topic which may require particular approaches to engage pregnant women, thereby limiting the generalisability of our findings. Although our interview sample was small, no new concepts arose before the last interview was concluded, giving us confidence that we sufficiently explored the special knowledge of our recruitment staff. Participating recruitment midwives and nurses provided an in-depth explanation of pregnant women’s barriers and enablers to towards research participation in general and we believe that these are largely transferrable to other settings.

## Conclusions

Recruitment of women in the antenatal setting poses particular logistical challenges requiring strategies to best manage the environment. While many pregnant women were interested in alcohol research for altruistic reasons, engagement with a sensitive topic such as alcohol use in pregnancy was facilitated by a non-judgmental and non-targeted approach. Influences such as privacy, distractions, partner’s opinion, time factors and level of clinical support either facilitated or hindered a woman’s engagement with the research. These findings will assist in the support and training of perinatal recruitment staff to optimise the informed decision making of women to participate in perinatal research.
